# Prevalence and impact of invasive fungal infections in intensive care

**DOI:** 10.1186/cc12026

**Published:** 2013-03-19

**Authors:** JM Patel, K Couper, T Melody, R O'Brien, D Parekh

**Affiliations:** 1 Heart of England NHS Foundation Trust, Birmingham, UK

## Introduction

Invasive fungal infections (IFI) affect 1% of ICU patients and are increasing in incidence. IFIs are associated with a poor prognosis, which is further complicated by difficulties in identification of fungal organisms by traditional culture methods and the emergence of Candida species resistant to triazole therapy [1,2]. This study aimed to assess the prevalence of IFIs, the organisms responsible and outcomes of patients affected.

## Methods

Patients admitted to the Heart of England NHS Foundation Trust ICUs who acquired an IFI were identified through the hospital Fungal Infection Risk Evaluation (FIRE) study database. All ICU patients admitted between October 2009 and March 2011 were used as a comparative cohort. Data collected included: baseline demographics, length of stay, ICU and hospital mortality, and nature of IFI. Data were analysed using Student's *t *test for continuous data and Fischer's exact test for categorical data.

## Results

A total of 2,426 patients were admitted to Heart of England NHS Foundation Trusts ICUs during the study period. Of these, 31 patients were identified as having an IFI (1.3%). Baseline demographic data were similar between groups. Patients with IFI had significantly longer ICU length of stay (19 days vs. 5 days, *P <*0.0001) and required more days of advanced organ support (12 days vs. 3 days, *P <*0.0001). A trend towards higher hospital mortality (41% vs. 27%, *P *= 0.08) was observed. *Candida albicans *was the predominant organism cultured (64%), followed by *Candida glabrata *(23%), with other Candida species accounting for the remaining 13% of IFIs. Sites of IFIs were blood (45%), intra-abdominal (39%), and pleural (16%). Most patients (52%) who acquired an IFI had had intra-abdominal surgery prior to ICU admission. The majority of patients (71%) were treated with echinocandins, whilst of the nine patients who were initially treated with flucanazole, six (67%) required therapy escalation to an echinocandin.

## Conclusion

The results of our study are consistent with other published data, in that whilst IFI prevalence is low, they are associated with increased morbidity in critically ill patients. This study has led to a change in hospital policy regarding antifungal use in the ICU, with echinocandins being first-line in the pre-emptive treatment of IFI. We keenly await the results of the FIRE study, which will provide important insights to identification of patients at risk of IFIs and optimal drug therapy.
